# Lower Rate of Cardiovascular Complications in Patients on Bolus Insulin Analogues: A Retrospective Population-Based Cohort Study

**DOI:** 10.1371/journal.pone.0079762

**Published:** 2013-11-07

**Authors:** Simona Cammarota, Lucio Marcello Falconio, Dario Bruzzese, Alberico Luigi Catapano, Manuela Casula, Anna Citarella, Luigi De Luca, Maria Elena Flacco, Lamberto Manzoli, Maria Masulli, Enrica Menditto, Andrea Mezzetti, Salvatore Riegler, Ettore Novellino, Gabriele Riccardi

**Affiliations:** 1 Center of Pharmacoeconomics and Drug Utilization (CIRFF), “Federico II” University of Naples, Naples, Italy; 2 Department of Preventive Medical Sciences, “Federico II” University of Naples, Naples, Italy; 3 Epidemiology and Preventive Pharmacology Centre (SEFAP), Department of Pharmacological Sciences, University of Milan, Milan, Italy; 4 Multimedica IRCCS, S.S. Giovanni, Milan, Italy; 5 Center for Pharmacoepidemiology, Department of Medicine, Karolinska Institutet, Stockholm, Sweden; 6 Section of Hygiene, Epidemiology, Pharmacology and Legal Medicine, "G. D'Annunzio" University Foundation, Chieti, Italy; 7 Department of Clinical and Experimental Medicine, “Federico II” University of Naples, Naples, Italy; 8 Clinical Research Centre, “G. D'Annunzio” University Foundation, Chieti, Italy; University of KwaZulu-Natal, South Africa

## Abstract

**Background:**

Few studies are available evaluating the impact of rapid-acting insulin analogues on long-term diabetes outcomes. Our aim was to compare the use of rapid-acting insulin analogues versus human regular insulin in relation to the occurrence of diabetic complications in a cohort of diabetic patients through the analysis of administrative databases.

**Methods:**

A population-based cohort study was conducted using administrative data from four local health authorities in the Abruzzo Region (900,000 inhabitants). Diabetic patients free of macrovascular disease at baseline and treated either with human regular insulin or rapid-acting insulin analogues were followed for a maximum of 3 years. The incidence of diabetic complications was ascertained by hospital discharge claims. Hazard ratios (HRs) and 95% CIs of any diabetic complication and macrovascular, microvascular and metabolic complications were estimated separately using Cox proportional hazard models adjusted for patients’ characteristics and anti-diabetic drug use. Propensity score matching was also used to adjust for significant difference in the baseline characteristics between the two treatment groups.

**Results:**

A total of 2,286 patients were included: 914 receiving human regular insulin and 1,372 rapid-acting insulin analogues. During the follow-up, 286 (31.3%) incident events occurred in the human regular insulin group and 235 (17.1%) in the rapid-acting insulin analogue group. After propensity score-based matched-pair analyses, rapid-acting insulin analogues users had a HR of 0.73 (0.58–0.92) for any diabetes-related complication and HRs of 0.73 (0.55–0.93) and 0.55 (0.32–0.96) for macrovascular and metabolic complications respectively, as compared with human regular insulin users. No difference between the two groups was found for microvascular complications.

**Conclusions:**

Our findings suggest that the use of rapid-acting insulin analogues is associated with a lower risk of cardiovascular and metabolic complications compared with human regular insulin use.

## Introduction

The major challenge in diabetes management is preventing or delaying the progression of diabetes-related complications. The landmark trials in type 1 [1,2] and type 2 [3,4] diabetic patients have demonstrated a strong inverse relationship between blood glucose levels and the occurrence of long-term microvascular complications. 

Recently an increasing body of evidence, not yet fully supported by randomized clinical trials, has suggested that postprandial hyperglycemia is directly implicated in cardiovascular complications [5-7]. The hypothesis is that a better control of postprandial glucose (PPG) levels may be an important therapeutic target in the prevention of cardiovascular complications in diabetic patients. 

The use of rapid-acting insulin analogues (Lispro, Aspart, Glulisine) provides lower postprandial glucose excursions in people with diabetes [8-11]. Nevertheless their effects on long-term outcomes are unknown. 

A recent observational study showed that the rapid-acting insulin analog gluslisine was associated with a reduced incidence of macro- and microvascular events in patients with type 2 diabetes in comparison with human regular insulin [12]. However, the primary care setting used in that study didn’t allow to capture all events but only those registered by the general practitioners (GPs). 

The aim of this study is therefore to compare the use of rapid-acting insulin analogues and human regular insulin in relation to the first occurrence of any diabetes-related complication in a cohort of diabetic patients without macrovascular disease at baseline through the analysis of administrative databases. 

## Methods

We conducted a retrospective, population-based cohort study using administrative data from four local health authorities in the Abruzzo Region of Central Italy, that comprise about 900,000 inhabitants (68% of the overall regional population). 

Details regarding data sources and study design have been published previously [13]. Briefly, a record-linkage analysis of outpatient drug prescriptions, hospital discharges, prescriptions for laboratory tests, services use and specialistic consultations and the civil registry was performed including data from January 1, 2005 to December 31, 2008. All information was linked through a unique and anonymous personal identification code. In order to protect the patient’s privacy the inverse process was prevented by the deletion of the conversion table. Because this automated system is anonymous, neither ethical committee approval nor informed consent was required. Furthermore the anonymous data file is routinely used by the regional health authorities for epidemiological and administrative purposes. Permission to use it for the present study was granted by the responsible authority.

### Study population

**Figure 1:**
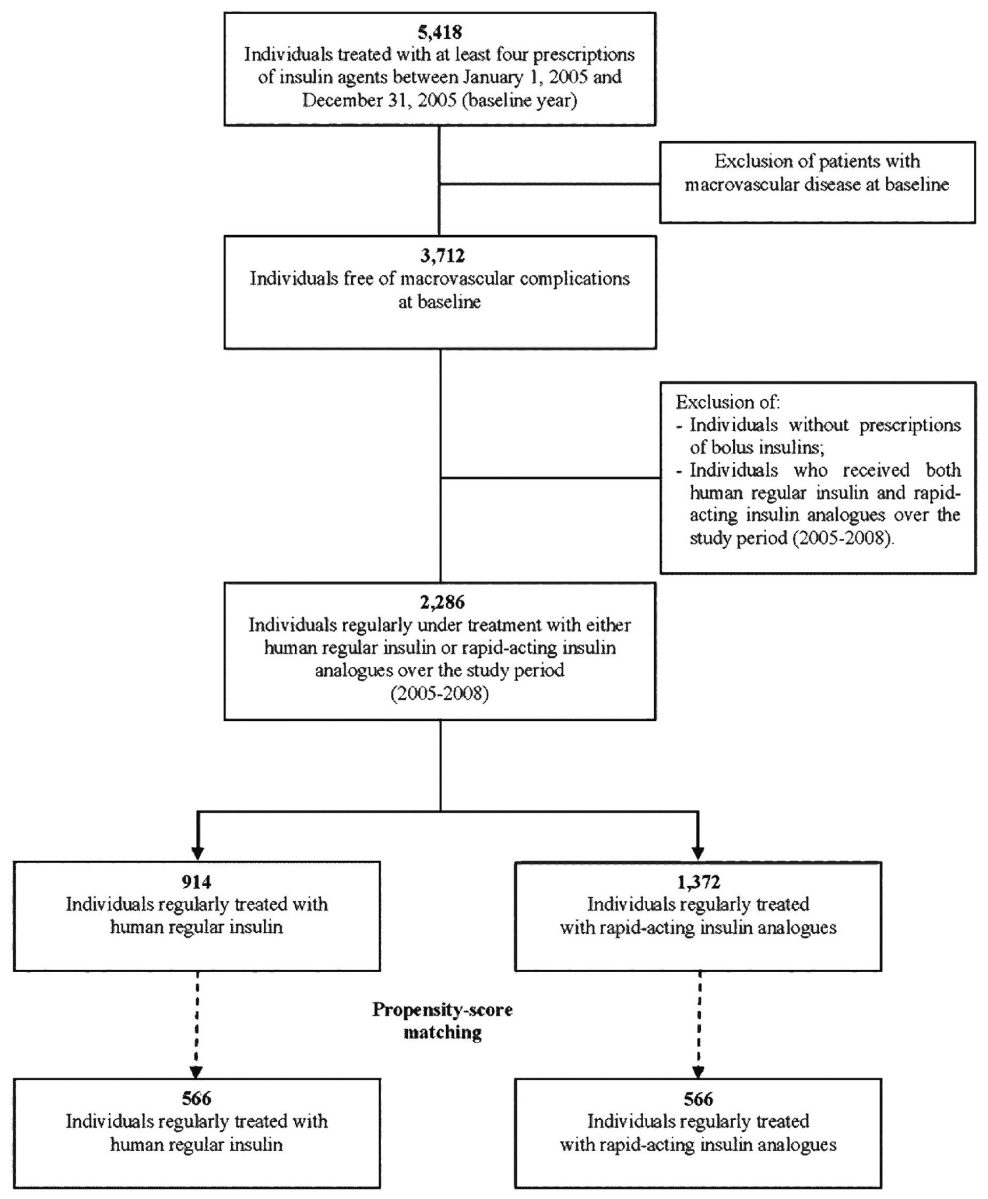
Patient selection flowchart.

A flow chart of the study population selection is depicted in Figure 1. From the source population we identified individuals with at least four prescriptions of insulin agents between January 1, 2005 and December 31, 2005 (baseline year). 

At baseline, we excluded patients if they had (i) at least one prescription of antiplatelets (excluding low-dose aspirin), pentoxifylline or nitrates, which are commonly used for the treatment of ischemic cardiovascular diseases, or a diagnosis of macrovascular disease in hospital discharge or procedure codes reported on prescriptions for services use [13]; (ii) no prescription of either human regular insulin or rapid-acting insulin analogues;(iii) prescriptions of both human regular insulin or rapid-acting insulin analogues during the whole study period (January 1, 2005 to December 31, 2008). Individuals who met the criteria were divided into two mutually exclusive groups (study cohorts): those treated with human regular insulin and those treated with rapid-acting insulin analogues throughout the entire follow-up. In both groups the bolus insulin was used alone or in combination with other types of insulin (premixed insulin and basal insulin) or oral hypoglycemic drugs, or both. 

Each individual accumulated person-years of follow-up from January 1, 2006 until the date of hospital admission for any diabetic complications, censoring (death or emigration), or December 31, 2008 (i.e. the end of the follow-up), whichever occurred first. 

### Covariates

For each patient the following potential confounders were assessed at baseline: age, sex, metabolic and microvascular complications, and concomitant drugs. The algorithm developed for identifying the microvascular and metabolic complications at baseline (reasonable proxy measure of severity and duration of diabetes) has been described elsewhere [13]. Prescriptions of other anti-diabetic drugs (intermediate/long-acting insulins, premixed insulins and different oral hypoglycemic agents), low-dose aspirin, antiarrhythmic agents, antihypertensive agents and lipid-lowering agents were also included. For each anti-diabetic agent (insulins and oral hypoglycemics) we further calculated the mean daily dose based on drug supplies through the total follow-up and categorized them into quartiles.

Finally, we also assessed the number of hemoglobin A1C tests performed by patients during the follow-up.

### Outcomes

The primary outcome of this study was the first occurrence of any diabetes-related complication during the follow-up. The secondary outcome was the first occurrence of specific clinical conditions including macrovascular, microvascular and metabolic complications [13]. To evaluate the risk of hospitalization we used only ordinary hospital admissions (i.e. day hospital admissions were excluded). Long-term complications were captured as an outcome of interest only if recorded as primary discharge diagnosis.

### Statistical analysis

The chi-square test (categorical variables) or unpaired t-test (continuous variable) for numerical variables were used to determine whether there were differences between baseline characteristics of patients treated with human regular insulin or rapid-acting insulin analogues.

Two statistical methods were used to evaluate the risk associated with bolus insulin treatment. First, we constructed Cox proportional hazards models to derive hazard ratios (HRs) and 95% confidence intervals (CIs), using the human regular insulin group as the reference group. Estimates were adjusted for age, gender, previous microvascular and metabolic complications, number of HbA1c tests, use of low-dose aspirin, antiarrhythmics agents, antihypertensive and lipid-lowering agents and mean daily dosage of anti-diabetic drugs in quartiles. Second, we performed a propensity-score (PS) matched Cox proportional hazard analysis. Specifically, we used PS analysis to identify subsamples of the two treatment groups (human regular insulin and the rapid-acting insulin analogues) that are “balanced” on all covariates listed in Table 1. This approach has been shown to be efficient for bias reduction due to the lack of randomization [14]. A flow diagram for identifying and creating the matched sample, together with the standardized differences in covariate means and prevalence between the two treatment groups, is available in the Supporting Information [see Figure S1; Figure S2]. 

**Table 1:** Baseline characteristics and propensity score matched baseline characteristics of patients treated with human regular insulin or rapid-acting insulin analogues.

	Unmatched cohorts			Propensity-score matched cohorts		
	Human regular insulin	Rapid-acting Insulin Analogues	*P*	Human regular insulin	Rapid-acting Insulin Analogues	*P*
N	914	1,372		566	566	
Age, years^a^	68.8±15.4	58.5±17.4	<0.0001	65.1 ± 16.2	65.3 ± 14.8	0.800
Female, n (%)	522 (57.1)	725 (52.8)	0.045	304 (53.7)	307 (54.2)	0.856
*Patients with 1 or more diabetic complications, n (%)*						
Metabolic	23 (2.5)	25 (1.8)	0.257	12 (2.1)	14 (2.5)	0.860
Microvascular	214 (23.4)	290 (21.1)	0.198	143 (25.3)	141 (24.9)	0.892
*Patients with at least 1 prescription of, n (%)*						
Low-dose aspirin	390 (42.7)	511 (37.2)	0.009	237 (41.9)	247 (43.6)	0.557
Antiarrhythmic agents	127 (13.9)	109 (7.9)	<0.0001	56 (9.9)	54 (9.5)	0.835
Antihypertensive agents	655 (71.7)	876 (63.8)	<0.0001	404 (71.4)	395 (69.8)	0.557
Lipid-lowering agents	213 (23.3)	409 (29.9)	0.001	159 (28.1)	169 (29.9)	0.504
*Other anti-diabetic drugs, n(%)*						
Intermediate/Long-acting insulin	379 (41.5)	448 (32.7)	<0.0001	280 (49.5)	270 (47.7)	0.534
Glargine	96 (10.5)	793 (57.8)	<0.0001	96 (17)	98 (17.3)	0.715
Premixed	345 (37.7)	171 (12.5)	<0.0001	110 (19.4)	117 (20.7)	0.531
Oral hypoglycemics	267 (29.2)	499 (36.4)	<0.0001	185 (32.7)	209 (36.9)	0.130
Metformin	241 (26.4)	420 (30.6)	0.027	165 (29.2)	167 (29.5)	0.899
Sulphonylureas	58 (6.3)	132 (9.6)	0.004	49 (8.7)	46 (8.1)	0.745
Thiazolidinediones	7 (0.8)	25 (1.8)	0.022	6 (1.1)	7 (1.2)	0.782
Other hypoglycemics	56 (6.1)	105 (7.7)	0.154	41 (7.2)	47 (8.3)	0.501
Average number of HbA1c tests per year^a^	0.7 ± 1.5	1.1 ± 1.3	<0.001	0.7 ± 1.1	0.8 ± 1.2	0.364

^a^ Data are mean ±SD.

Kaplan-Meier estimates were used to generate time to event curves for the primary- and secondary outcomes. In the unmatched sample, survival curves were compared across the two treatment groups by the log-rank test. In the matched sample, survival curves were constructed using paired Kaplan-Meier estimates and compared using the Klein-Moeschberger test.

All analyses were performed using R (A Language and Environment for Statistical Computing, Release 2.14.1; R Foundation for Statistical Computing, http://www.R-project.org) and SPSS software version 17.1 for Windows (SPSS Inc, Chicago, IL, USA). 

## Results

### Baseline characteristics

Overall, 2,286 patients were included into the analyses: 914 in the human regular insulin group and 1,372 in the rapid-acting insulin analogue group. The total number of patient-years accumulated was 2,084 for the human regular insulin users and 3,675 for the rapid-acting insulin analogue users.

As shown in Table 1, the two study groups had different characteristics at baseline. Compared with patients in the rapid-acting insulin analogue group, those in the human regular insulin group were older, more likely to be women and more likely to receive low-dose aspirin, antihypertensive drugs and antiarrhythmic agents but less likely to receive lipid lowering agents and oral hypoglycemic agents (metformin, sulphonylureas, thiazolidenediones) and less likely to perform HbA1c tests. The mean daily dosage for all insulin formulations was 1.13±0.64 SD in the rapid-acting insulin analogue group vs. 1.03±0.80 SD in the human regular insulin group (p=0.001). For oral hypoglycemic, the mean daily dosage was 0.72±0.68 SD in the rapid-acting insulin analogue group vs. 0.58±0.54 in the human regular insulin group (p=0.002) At baseline, no difference was found between the two groups in the prevalence of microvascular and metabolic complications. 

Using the PS approach, 566 patients treated with human regular insulin were matched with an equal number of patients receiving rapid-acting insulin analogues. Baseline characteristics were balanced for all covariates in these matched cohorts (Table 1). No difference between the two PS matched cohort was found for the mean daily dose of insulin and oral hypoglycemic agents.

### Follow-up

**Figure 2:**
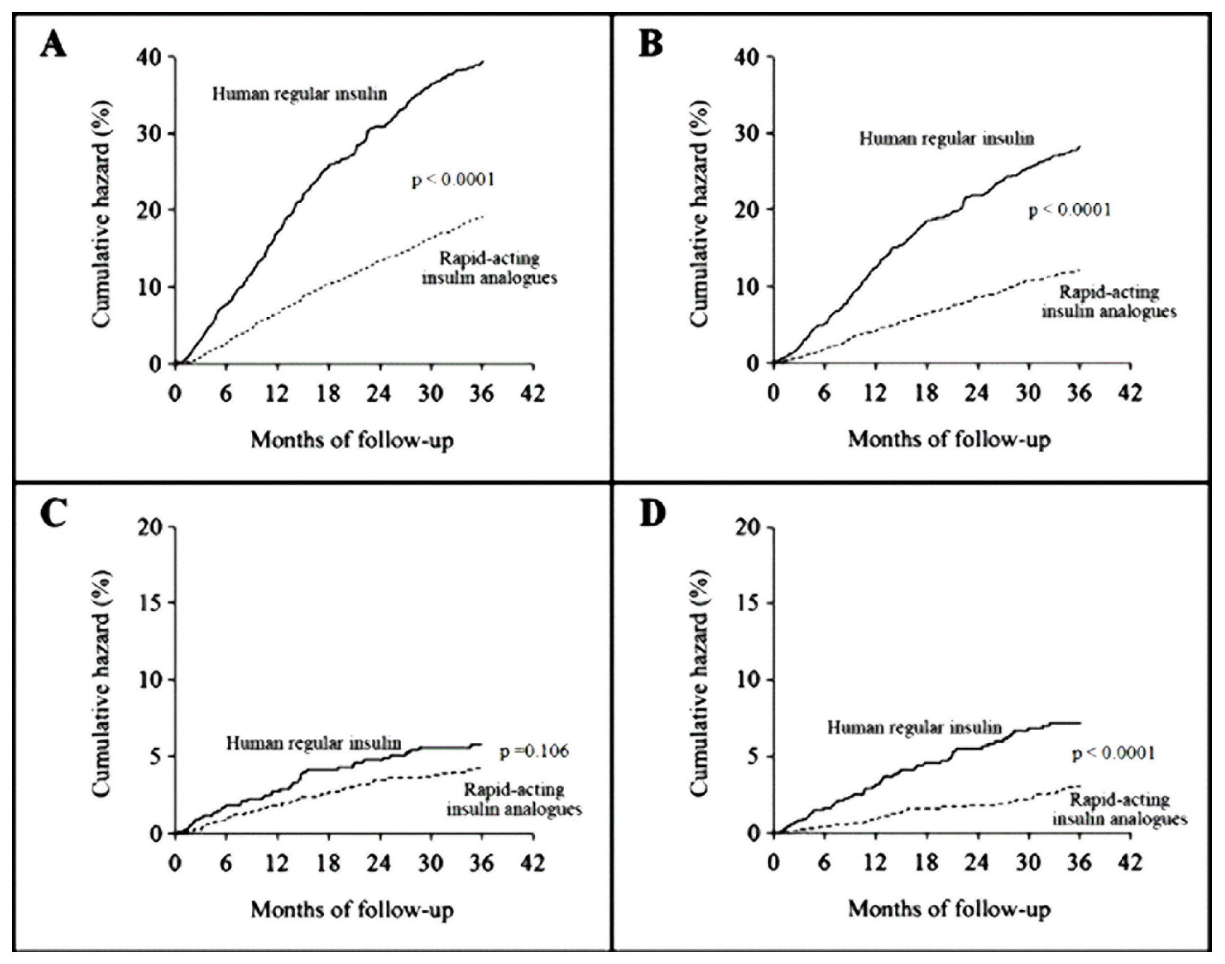
Cumulative hazard according to the two treatment group of (A) Any diabetes-related complications (B) Macrovascular complications, (C) Microvascular complications and (D) Metabolic complications.

During the 3-year follow-up, there were 286 incident events (31.3%) in the human regular insulin group and 235 (17.1%) in the rapid-acting insulin analogue group, with a difference between the two treatment groups in the cumulative HR for any diabetic complications that increased over time (p<0.0001 by the log-rank test) (Figure 2). 

After multivariable adjustments, using human regular insulin group as the reference group, the HR was 0.56 (95% CI: 0.45-0.69) for any diabetic complications in the rapid-acting insulin analogue group. Regardless of secondary outcomes, we found 203 macrovascular events (22.2%) in the human regular insulin group and 147 (10.7%) in the rapid-acting insulin analogue group, with a significant reduction in macrovascular complications associated with the use of rapid-acting insulin analogues (HR=0.56; 95% CI:0.43-0.72) (Table 2). Among macrovascular events, a highly significant reduction was observed for each specific clinical condition in the rapid-acting insulin analogue users. A statistically significant difference was also found in the risk of metabolic complications. Among these, only the difference in hyperglycemic events remained significant after multivariate adjustments. Conversely, no difference between the two groups was found for microvascular disease rates (Table 2).

**Table 2:** Cumulative incidence and Cox Proportional Hazard Ratios (HRs) of diabetes-related complications in patients treated with human regular insulin or a rapid-acting insulin analogue.

	Unmatched cohorts				Propensity-score matched cohorts		
Complications	Human regular insulin *n* (%)	Rapid-acting Insulin Analogues *n* (%)	Adjusted HR^a^ (95% CI)	Human regular insulin *n* (%)		Rapid-acting Insulin Analogues *n* (%)	HR (95% CI)
Any complication	286 (31.3)	235 (17.1)	0.56 (0.45-0.69)	165 (29.1)		132 (23.3)	0.73 (0.58-0.92)
Macrovascular	203 (22.2)	147 (10.7)	0.56 (0.43-0.72)	110 (19.4)		87 (15.4)	0.73 (0.55-0.95)
Cardiovascular disease	107 (11.7)	95 (6.9)	0.62 (0.44-0.86)	63 (11.1)		53 (9.4)	0.77 (0.54-1.10)
Peripheral vascular disease	21 (2.3)	14 (1.0)	0.32 (0.14-0.75)	14 (2.5)		8 (1.4)	0.53 (0.22-1.27)
Cerebrovascular disease	75 (8.2)	38 (2.8)	0.55 (0.35-0.88)	33 (5.8)		26 (4.6)	0.73 (0.44-1.20)
Microvascular	43 (4.7)	53 (3.9)	0.73 (0.45-1.20)	27 (4.8)		26 (4.6)	0.89 (0.54-1.48)
Metabolic	52 (5.7)	37 (2.7)	0.36 (0.21-0.59)	35 (6.2)		21 (3.7)	0.55 (0.32-0.94)
Hyperglycemia	34 (3.7)	21 (1.5)	0.36 (0.21-0.59)	21 (3.7)		11 (1.9)	0.48 (0.23-0.99)
Hypoglycemia	19 (2.1)	16 (1.2)	0.51 (0.23-1.12)	14 (2.5)		12 (2.1)	0.62 (0.29-1.34)

IA= Insulin Analogues. Human regular insulin reference group. ^a^ Adjusted for variables reported in Table 1 plus mean daily dosage of both insulin and oral hypoglycemic agents in quartiles.

The PS matched Cox models supported these results (Figure S3). Specifically, the differences between the two groups in relation to the risk of any diabetic (HR=0.73; 95% CI:0.58-0.92), macrovascular (HR=0.73; 95% CI:0.55-0.95) and metabolic complications (HR=0.55; 95% CI:0.32-0.94) remained statistically significant. However among macrovascular events, no difference between the two groups was found for any specific clinical condition. The analysis confirmed the lack of statistical significance for the difference in microvascular complication rates (HR=0.89; 95% CI:0.54-1.48) (Table 2). 

### Supplementary analyses

Because we have previously shown that the use of insulin glargine was associated with a lower risk of diabetic complications than intermediate- and long-acting human insulins [13], we repeated all analyses on the subcohort of patients who did not receive any prescription of insulin glargine throughout the entire study period. Baseline characteristics of the unmatched and matched subcohorts are available in Table S1.

For all endpoints, results were similar to those presented for the entire study population. In particular in the unmatched subcohorts, the adjusted HR estimates were 0.57 (95% CI: 0.46-0.72) for any diabetic complication, 0.57 (95% CI: 0.43-0.76) for macrovascular complications and 0.39 (95% CI: 0.22-0.69) for metabolic complications in rapid-acting insulin users as compared with those on human regular insulin; no statistical significant difference was found between the two subgroups in relation to microvascular event rates (Table S2). All results were confirmed after PS matched-pair analyses (Table S2).

## Discussion

The major finding of our study is that patients given rapid-acting insulin analogues developed fewer diabetic complications (especially, fewer macrovascular events) than those given human regular insulin. 

Due to the lack of randomization, significant differences in baseline characteristics exist between the two treatment study group. Patients on human regular insulin are expected to be at higher risk of developing cardiovascular complications than those on rapid-acting insulin analogue, because they were older and more likely to use low-dose aspirin and antiarrhythmic and antihypertensive agents. On the other hand, patients treated with bolus insulin analogues were more often men, reported a higher number of HbA1c tests and a higher mean daily dose of all anti-diabetic agents than patients receiving human regular insulin. Despite these differences between the two study cohorts, the reduction of both primary and secondary outcomes remained significant even after adjusting for these and other potential confounders evaluated in this study. These results were also supported by PS matched-pair analyses performed on the two balanced subsamples.

Moreover, based on our previous finding that a lower risk of any diabetic complication was associated with the use of insulin glargine [13] we carried out the analyses on the subcohorts of patients who did not receive any prescription of insulin glargine during the whole study period in order to evaluate any possible interference of glargine use on the study results. In this subgroup analysis, a statistically significant reduction for any diabetes-related complication was still observed in patients treated with rapid-acting insulin analogues. Similarly, macrovascular and metabolic events were markedly decreased in patients given rapid-acting insulin analogues as compared with those receiving human regular insulin.

Our findings were in line with a previous observation suggesting that the use of rapid-acting insulin analogue glulisine could be associated with a risk reduction of 24% for macrovascular events in patients with type 2 diabetes as compared with the use of human regular insulin [12]. In that study, the assessment of the outcomes was based on ICD codes registered by GPs only. The relevance of our study is the use of a large-scale, population-based cohort and the ability to draw information from a real-world setting through the analysis of administrative data. As a matter of fact, the majority of diabetes-related trials do not focus on significant cardiovascular outcomes and are performed in study groups that are not representative of the general diabetic population [15]. We believe that the strength of administrative data for our study question is in the ability to capture all acute diabetes complication events that is extremely important for the assessment of the quality of care in the real-world. 

Nevertheless, we acknowledge that an observational study using administrative data is prone to potential biases. In particular, our findings may be subject to confounding by indication due to the lack of randomization. In fact the allocation of treatment is based on the clinical judgment of physicians and may be related to a different cardiovascular risk profile between the two groups. However, all PS matching resulted in full balance of all patients characteristics and is likely to have minimized confounding by indication enhancing the comparability of patients. In addition, Cox regression analyses were conducted with macrovascular complications as the endpoint after stratification for gender or age (<65 years versus ≥65 years); the results remained essentially unchanged when the association between study treatment and macrovascular events was examined in the different subgroups (Table S3).

The presence of unrecognized confounders could lead us to over-estimate the magnitude of the association between exposure and outcome as compared with the results of randomized clinical trials. The analysis of confounders that were included in the Cox-models was limited to variables collected by administrative data. A good deal of information on the degree of metabolic control, severity and duration of diabetes and on lifestyle attitudes, which are known to influence the likelihood of developing complications [16], is not available in our data sources. Nevertherless, we attempted to limit the role of these confounders by adjusting for age, sex, concomitant drugs and the presence of microvascular or metabolic events at baseline (the latter two factors may be a proxy of duration and severity of diabetes); moreover we reported all analyses in the two cohorts after PS matching.

Another limitation of our study is that our database does not allow us to ascertain with confidence the type of diabetes as a potential confounder. The higher proportion of younger patients in the rapid-acting insulin analogue group suggests the possibility of a higher prevalence of patients with type 1 diabetes. Therefore, we attempted to explore the effect of this variable on our results by using the following criteria for identifying type 1 diabetes cases: no records of oral hypoglycemic agents, specific ICD9 code for type 1 diabetes (250.x1 or 250.x3), diabetic ketoacidosis episode (250.x1), age<40 year [17]. According to this analysis, the percentage of type 1 diabetic patients was 5.6% in the human regular insulin group and 15.7% in the rapid-acting insulin analogue group. However, our results remain consistent after the inclusion of this variable in all models or after the exclusion of type 1 patients from the analyses (data not shown).

Our findings could be explained by a better quality of care in the group treated with bolus insulin analogues that could be associated with a more favorable prognosis in that group [18]. However, we also adjusted for the number of HbA1c tests as a proxy for the quality of diabetes care in all models.

Additionally, our study population included diabetic patients free of macrovascular diseases at baseline, who may have been misclassified because of medical errors in the medication and diagnosis codes or the short baseline period (1 year). However, this type of error does not seem to be very high because the cohort of diabetic patients with macrovascular disease at baseline represented about 30% of the overall sample, which is consistent with results from previous landmark studies [2,3]. Finally, because outcome data were drawn from hospital discharges, these data can be misclassified. However, outcome misclassification can be expected to occur independently of the insulin exposure under study and therefore a non-differential misclassification would have biased the results of the study towards the null hypothesis [19].

Despite these intrinsic limitations of observational data, several speculative explanations for our findings should be considered. In particular there are three demonstrated clinical effects of the rapid-acting insulin analogues that could explain why they may be more effective than the human regular insulin in reducing the risk of diabetic complications.

1. Overall glycemic control may be better with rapid-acting insulin analogues than with human regular insulin. These agents can be injected immediately before the meals, whereas human regular insulin should be injected 30-45 min before the meals [20]. In reality, most patients do not follow the strict time injection required for the human regular insulin administration and therefore in many cases the PPG levels are not optimally controlled with this agent [19,20]. In a randomized crossover study Annuzzi et al [21] showed that insulin lispro, in combination with neutral protamine Hagedorn (NPH) insulin, significantly reduced HbA1c (0.2%) in type 1 diabetic patients. Furthermore, in a large-scale multicentre trial Hermansen et al [22] demonstrated that the combination of the basal-bolus regimen (insulin detemir and insulin aspart) provided a significant reduction in HbA1c by 0.22% when compared with traditional insulins (NPH and human regular insulin) in patients with type 1 diabetes after 18 weeks of treatment. More recently, in a meta-analysis Mannucci et al [23] showed that the use of rapid-acting insulin analogues in type 2 diabetic patients provides a small but statistically significant reduction of HbA1c in comparison with human regular insulin. In our opinion the magnitude of the HbA1c reduction found in these studies in patients treated with rapid-acting insulin analogues is not sufficient to explain our findings.
2. Another plausible account might be that the greater beneficial effect of bolus insulin analogues on the reduction of diabetic complications (particularly macrovascular events), might be also attributable to their ability to better control PPG excursions. In line with those results, the Diabetes Intervention Study (DIS) [24] and The San Luigi Gonzaga Diabetes Study [25] demonstrated the importance of postprandial blood glucose control in preventing cardiovascular events in patients with type 2 diabetes. Conversely, the HEART2D trial [26] did not show any beneficial effect of specifically treating postprandial hyperglycemia in relation to the risk of cardiovascular events in diabetic patients. However, a post-hoc subgroup analysis of the HEART2D on a specific population (older patients) has found a lower risk of a subsequent cardiovascular events associated with the use of prandial insulin lispro [27]. In the light of these findings, the role of postprandial hyperglycemia in the development or progression of diabetic complication remains a sensitive and controversial issue. Our results refer to a cohort of diabetic patients without a recent cardiovascular event; in this respect they don’t resemble the profile of the HEART2D cohort. Unfortunately, the potential role of PPG levels in reducing the risk of developing long-term diabetes outcomes cannot be evaluated in our study given the lack of information on metabolic parameters in the database.
3. Finally, several studies have also shown a lower occurrence of hypoglycemia with the use of rapid-acting insulin analogues [28,29]. In our study we found a lower risk of metabolic events in the rapid-acting insulin analogue group. However, the incidence of hypoglycemic events, when analyzed separately, was not statistically different after adjusting for the covariates. Only hypoglycemic episodes requiring hospitalization were taken into account in our analyses and therefore it is possible that we underestimated the overall incidence of hypoglycemic episodes and the potential difference between the two treatment groups in that these conditions are more likely to be treated in an outpatient setting [30,31]. Hypoglycemic events have been recently recognized as important markers of cardiovascular risk in diabetic patients [32].

## Conclusions

This analysis from a real-world setting suggests that the use of rapid-acting insulin analogues, in comparison with human regular insulin, is associated with a lower risk of diabetic complications and particularly macrovascular events. Future trials may be required to determine the potential long-term clinical benefits of bolus insulin analogues in preventing cardiovascular diseases in diabetic patients.

## Supporting Information

Figure S1 **Flow chart for creating and validating propensity score-matched pairs.**
(PDF)

Figure S2 **Absolule standardized differences in covariates between the two study treatment groups, before and after propensity score matching.**
(PDF)

Figure S3 **Cumulative hazard according to the two treatment group matched for propensity score of (A) Any diabetes-related complications (Klein-Moeschberger for comparison, p=0.003), (8) Macrovascular complications (Klein- Moeschberger for comparison, p=0.003), (C) Microvascular complications (Klein-Moeschberger for comparison, p=0.853) and (D) Metabolic complications (Klein-Moeschberger for comparison, p=0.018).**
(PDF)

Table S1 **Baseline characteristics and propensity score matched baseline characteristics of study sub-cohorts without prescription of insulin glargine treated with human regular insulin or a rapid-acting insulin analogues.**
(PDF)

Table S2 **Cumulative incidence and Cox Proportional Hazard Ratios (HRs) of diabetes-related complications in patients without prescription of insulin glargine and treated with human regular insulin or a rapid-acting insulin analogue.**
(PDF)

Table S3 **Cumulative incidence and Cox Proportional Hazard Ratios (HRs) of Macrovascular complications in patients treated with human regular insulin or a rapid-acting insulin analogue stratified by Gender or Age.**
(PDF)
