# Supplementary figures and images for: Lower Rate of Cardiovascular Complications in Patients on Bolus Insulin Analogues: A Retrospective Population-Based Cohort Study
Source: PLoS One. 2013 Nov 7;8(11):e79762. doi: 10.1371/journal.pone.0079762 (PMC3820645; doi:10.1371/journal.pone.0079762)

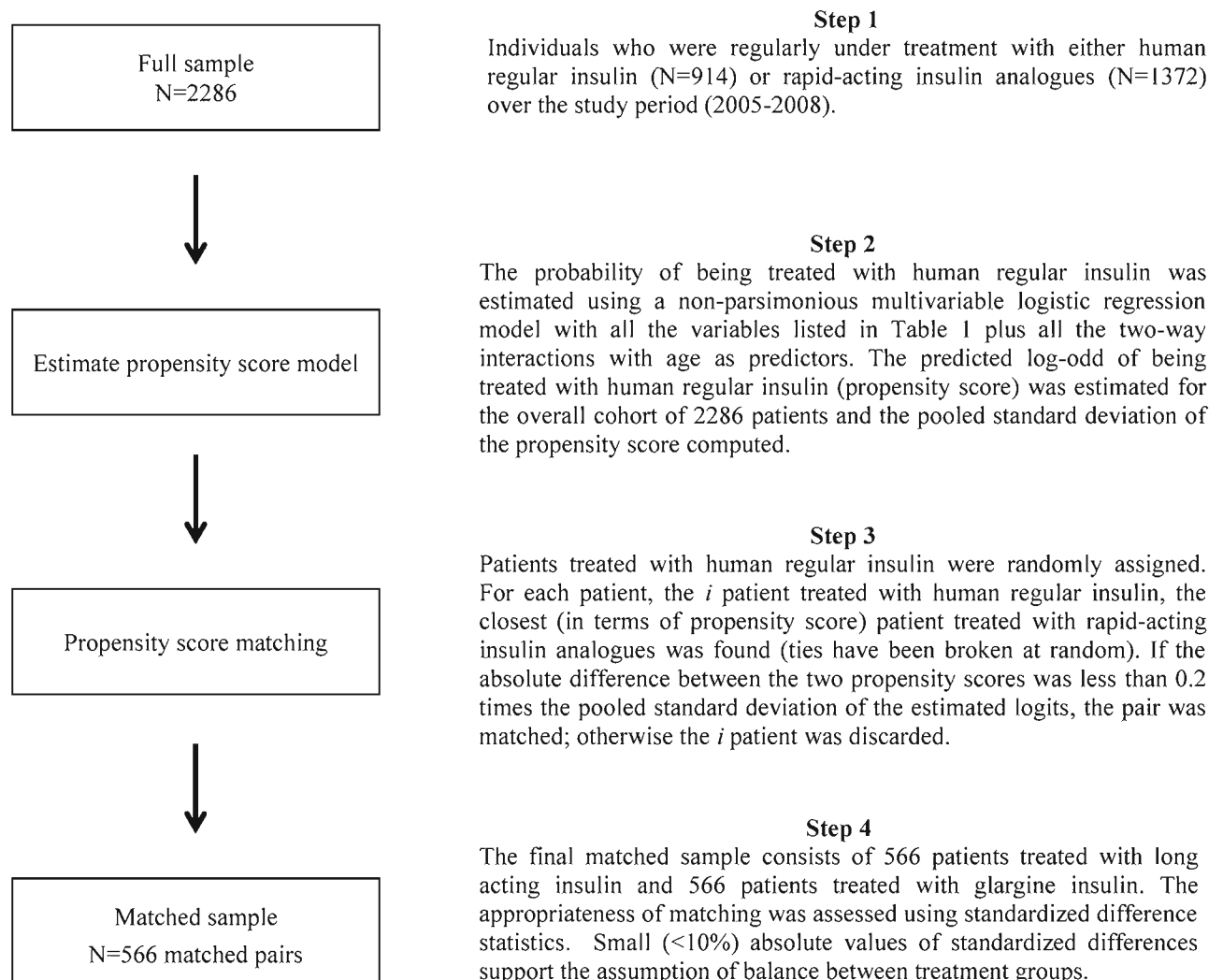

**Figure S1:** Flow chart for creating and validating propensity score-matched pairs

Supplement: Figure S1 — Flow chart for creating and validating propensity score-matched pairs. (PDF) [file pone.0079762.s001.pdf]
