# Supplementary material for: Lower Rate of Cardiovascular Complications in Patients on Bolus Insulin Analogues: A Retrospective Population-Based Cohort Study
Source: PLoS One. 2013 Nov 7;8(11):e79762. doi: 10.1371/journal.pone.0079762 (PMC3820645; doi:10.1371/journal.pone.0079762)
Supplement: Figure S2 — Absolule standardized differences in covariates between the two study treatment groups, before and after propensity score matching. (PDF) [file pone.0079762.s002.pdf]

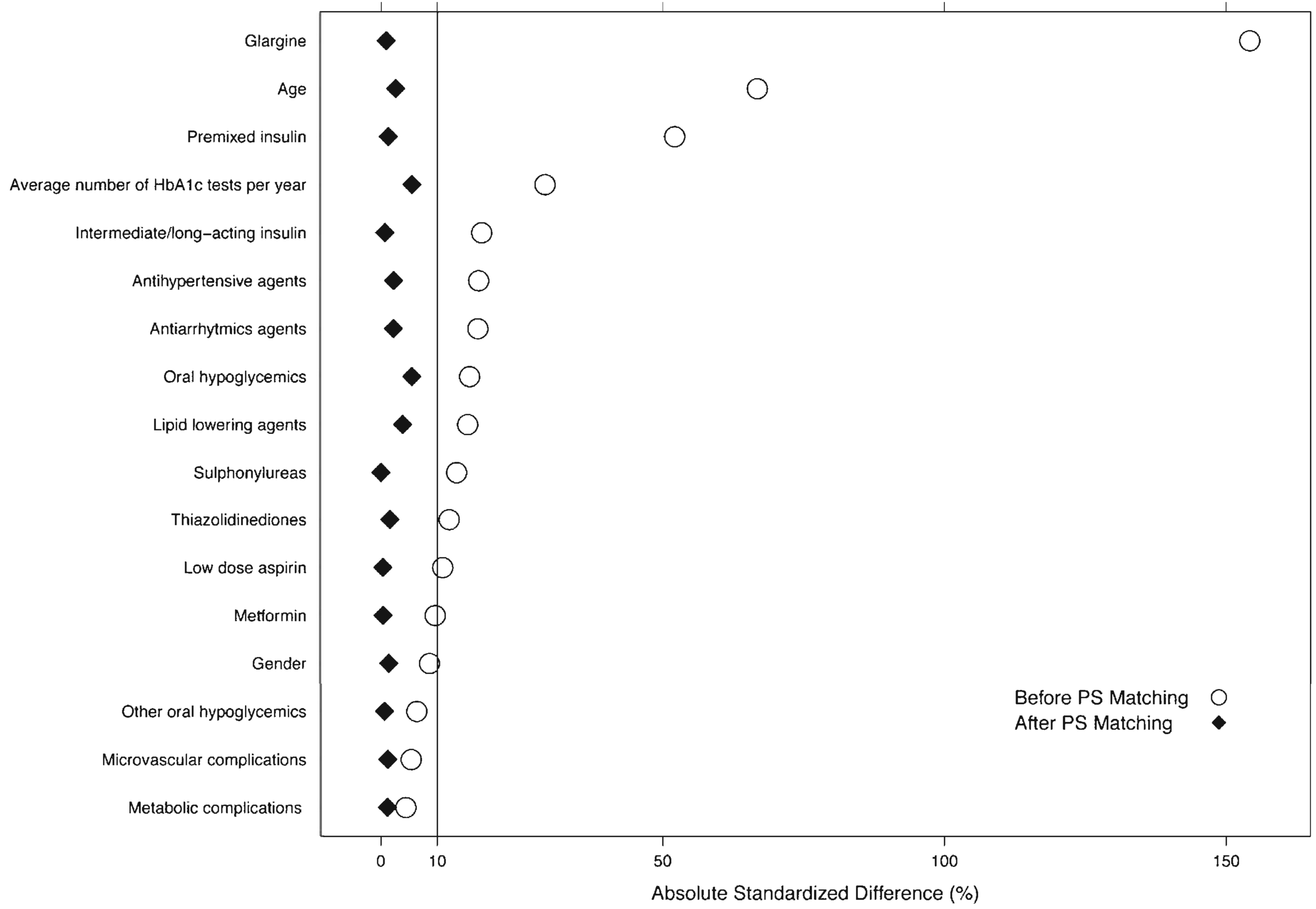

Figure S2. Absolute standardized differences in covariates between the two study treatment groups, before and after propensity score matching
