# Supplementary material for: Lower Rate of Cardiovascular Complications in Patients on Bolus Insulin Analogues: A Retrospective Population-Based Cohort Study
Source: PLoS One. 2013 Nov 7;8(11):e79762. doi: 10.1371/journal.pone.0079762 (PMC3820645; doi:10.1371/journal.pone.0079762)
Supplement: Figure S3 — Cumulative hazard according to the two treatment group matched for propensity score of (A) Any diabetes-related complications (Klein-Moeschberger for comparison, p=0.003), (8) Macrovascular complications (Klein- Moeschberger for comparison, p=0.003), (C) Microvascular complications (Klein-Moeschberger for comparison, p=0.853) and (D) Metabolic complications (Klein-Moeschberger for comparison, p=0.018). (PDF) [file pone.0079762.s003.pdf]

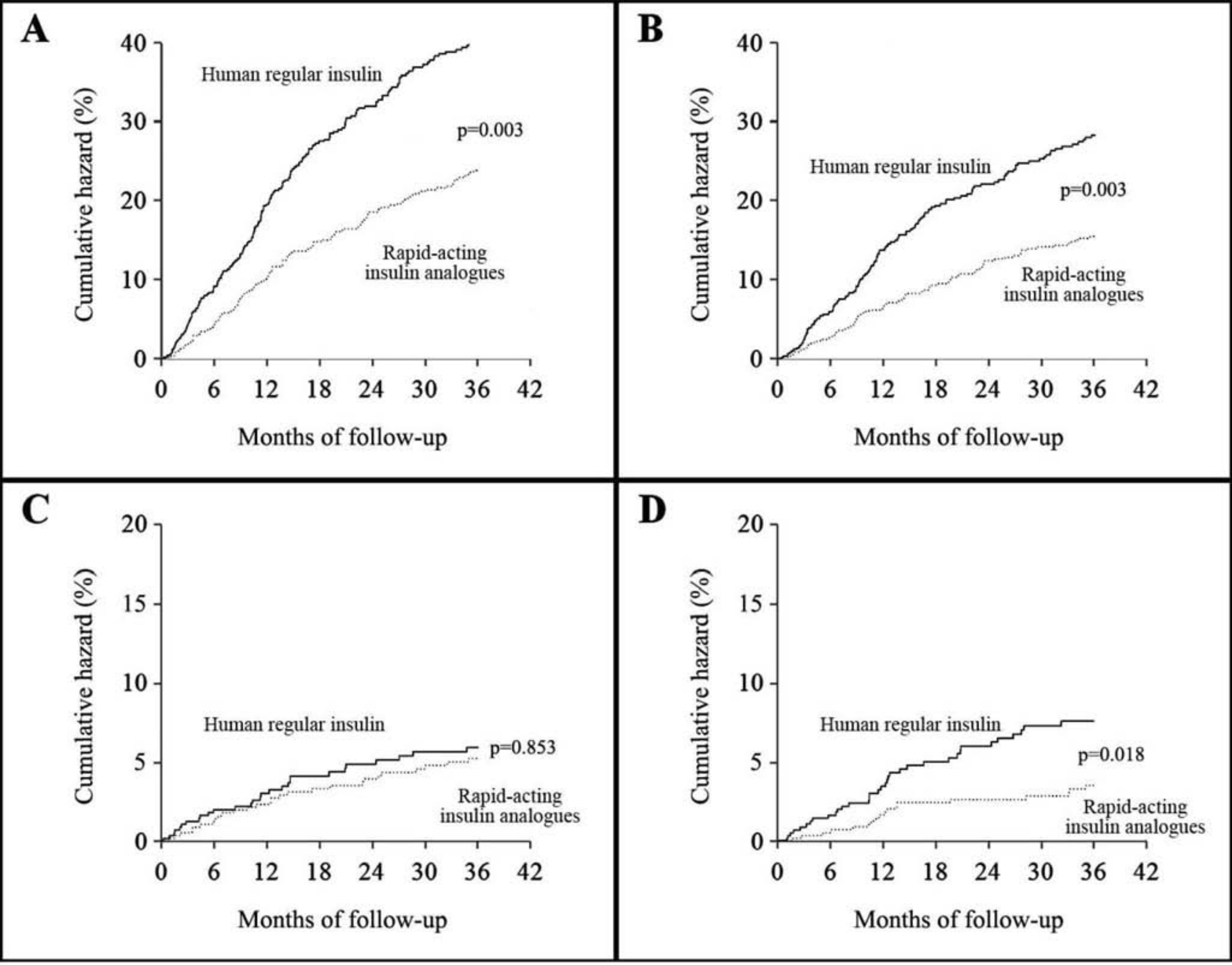

Figure S3: Cumulative hazard according to the two treatment group matched for propensity score of (A) Any diabetes-related complications (Klein-Moeschberger for comparison, p=O.003), (8) Macrovascular complications (Klein- Moeschberger for comparison, p=O.003), (C) Microvascular complications (Klein-Moeschberger for comparison, p=O.853) and (O) Metabolic complications (Klein-Moeschberger for comparison, p=O.018).
