# Supplementary material for: Lower Rate of Cardiovascular Complications in Patients on Bolus Insulin Analogues: A Retrospective Population-Based Cohort Study
Source: PLoS One. 2013 Nov 7;8(11):e79762. doi: 10.1371/journal.pone.0079762 (PMC3820645; doi:10.1371/journal.pone.0079762)
Supplement: Table S1 — Baseline characteristics and propensity score matched baseline characteristics of study sub-cohorts without prescription of insulin glargine treated with human regular insulin or a rapid-acting insulin analogues. (PDF) [file pone.0079762.s004.pdf]

Table S1: Baseline characteristics and propensity score matched baseline characteristics of study sub-cohorts without prescription of insulin glargine treated with human regular insulin or a rapid-acting insulin analogues

	Unmatched cohorts			Propensity-score mached cohorts		
	Human regular insulin	Rapid-acting insulin analogues	*P*	Human regular insulin	Rapid-acting insulin analogues	*P*
N	818	579		484	484	
Age, years[a]	69.76 ± 15.11	63.35 ± 16.55	<0.001	65.73 ± 16.06	65.07 ± 15.39	0.425
Female, n (%)	463 (56.6)	317 (54.7)	0.493	243 (50.2)	260 (53.7)	0.251
Patients with 1 or more diabetic complications, n (%)						
Metabolic	17 (2.1)	11 (1.9)	0.813	10 (2.1)	8 (1.7)	0.637
Microvascular	189 (23.1)	141 (24.4)	0.590	117 (24.2)	115 (23.8)	0.880
Patients with a t le ast 1 prescription of, n (%)						
Low-dose aspirin	355 (43.4)	238 (41.1)	0.393	205 (42.4)	199 (41.1)	0.680
Antiarrhythmic agents	114 (13.9)	52 (9)	0.004	46 (9.5)	48 (9.9)	0.831
Antihypertensive agents	591 (72.2)	403 (69.6)	0.285	337 (69.6)	334 (69)	0.835
Lipid-lowering agents	183 (22.4)	178 (30.7)	<0.001	138 (28.5)	135 (27.9)	0.825
Other anti-diabetic drugs, n(%)						
Intermediate/Long-acting insulin	356 (43.5)	314 (54.2)	<0.001	271 (56)	264 (54.5)	0.649
Premixed	332 (40.6)	105 (18.1)	<0.001	98 (20.2)	104 (21.5)	0.532
Oral hypoglycemics	225 (27.5)	221 (38.2)	<0.001	165 (34.1)	163 (33.7)	0.883

[a]Data are mean ±SD
