# Supplementary material for: Lower Rate of Cardiovascular Complications in Patients on Bolus Insulin Analogues: A Retrospective Population-Based Cohort Study
Source: PLoS One. 2013 Nov 7;8(11):e79762. doi: 10.1371/journal.pone.0079762 (PMC3820645; doi:10.1371/journal.pone.0079762)
Supplement: Table S2 — Cumulative incidence and Cox Proportional Hazard Ratios (HRs) of diabetes-related complications in patients without prescription of insulin glargine and treated with human regular insulin or a rapid-acting insulin analogue. (PDF) [file pone.0079762.s005.pdf]

Table S2. Cumulative incidence and Cox Proportional Hazard Ratios (HRs) of diabetes-related complications in patients without prescription of insulin glargine and treated with human regular insulin or a rapid-acting insulin analogue.

Complications	Unmatched cohorts			Propensity-score mached cohorts		
	Human regular insulin *n* (%)	Rapid-acting insulin analogues *n* (%)	Adjusted HR[a] (95% CI)	Human regular insulin *n* (%)	Rapid-acting insulin analogues *n* (%)	HR (95% CI)
Any complication	266 (32.5)	125 (21.6)	0.59 (0.47-0.74)	159 (32.9)	107 (22.1)	0.62 (0.48-0.79)
Macrovascular	190 (23.2)	83 (14.3)	0.59 (0.45-0.77)	105 (21.7)	70 (14.5)	0.62 (0.46-0.84)
Cardiovascular disease	102 (12.5)	48 (8.3)	0.61 (0.42-0.88)	64 (13.2)	38 (7.9)	0.55 (0.37-0.83)
Peripheral vascular disease	17 (2.1)	8 (1.4)	0.59 (0.24-1.46)	8 (1.7)	7 (1.4)	0.79 (0.28-2.20)
Cerebrovascular disease	71 (8.7)	27 (4.7)	0.57 (0.36-0.92)	33 (6.8)	25 (5.2)	0.71 (0.41-1.21)
Microvascular	42 (5.1)	24 (4.1)	0.66 (0.39-1.13)	29 (6)	23 (4.8)	0.73 (0.42-1.25)
Metabolic	46 (5.6)	20 (3.5)	0.43 (0.25-0.75)	31 (6.4)	15 (3.1)	0.44 (0.24-0.80)
Hyperglycemia	30 (3.7)	11 (1.9)	0.36 (0.17-0.75)	20 (4.1)	7 (1.4)	0.31 (0.13-0.74)
Hypoglycemia	16 (2)	9 (1.6)	0.56 (0.24-1.33)	11 (2.3)	8 (1.7)	0.67 (0.28-1.61)

Human regular insulin reference group. [a]Adjusted for variables reported in Table 1 plus mean daily dosage of both insulin and oral hypoglycemic agents in quartiles.
