# Supplementary material for: Lower Rate of Cardiovascular Complications in Patients on Bolus Insulin Analogues: A Retrospective Population-Based Cohort Study
Source: PLoS One. 2013 Nov 7;8(11):e79762. doi: 10.1371/journal.pone.0079762 (PMC3820645; doi:10.1371/journal.pone.0079762)
Supplement: Table S3 — Cumulative incidence and Cox Proportional Hazard Ratios (HRs) of Macrovascular complications in patients treated with human regular insulin or a rapid-acting insulin analogue stratified by Gender or Age. (PDF) [file pone.0079762.s006.pdf]

Table S3: Cumulative incidence and Cox Proportional Hazard Ratios (HRs) of Macrovascular complications in patients treated with human regular insulin or a rapid-acting insulin analogue stratified by Gender or Age.

	Unmatched cohorts		
	Human regular insulin *n* (%)	Rapid-acting insulin analogues *n* (%)	Adjusted HR[a] (95% CI); p
Age < 65 years (n=1059)	41 (14.6)	54 (6.9)	0.44 (0.27 – 0.71); p<0.001
Age ≥ 65 years (n=1227)	162 (25.6)	93 (15.7)	0.59 (0.44 – 0.79); p<0.001
Male (n= 1039)	95 (24.2)	75 (5.8)	0.46 (0.32-0.66); p<0.001
Female (n= 1247)	108 (20.7)	72 (9.9)	0.61 (0.44-0.89); p= 0.008

Human regular insulin reference group. [a]Adjusted for variables reported in Table 1 plus mean daily dosage of both insulin and oral hypoglycemic agents in quartiles.
